# Exploring the use of web searches for risk communication during COVID-19 in Germany

**DOI:** 10.1038/s41598-021-85873-4

**Published:** 2021-03-19

**Authors:** Kaja Kristensen, Eva Lorenz, Jürgen May, Ricardo Strauss

**Affiliations:** 1 Department of Medical Psychology, University Medical Center Hamburg-Eppendorf, Hamburg, Germany; 2 Department of Infectious Disease Epidemiology, Bernhard Nocht Institute for Tropical Medicine, Hamburg, Germany

**Keywords:** Diseases, Health care

## Abstract

Risk communication during pandemics is an element of utmost importance. Understanding the level of public attention—a prerequisite for effective communication—implicates expensive and time-consuming surveys. We hypothesise that the relative search volume from Google Trends could be used as an indicator of public attention of a disease and its prevention measures. The search terms ‘RKI’ (Robert Koch Institute, national public health authority in Germany), ‘corona’ and ‘protective mask’ in German language were shortlisted. Cross-correlations between these terms and the reported cases from 15 February to 27 April were conducted for each German federal state. The findings were contrasted against a timeline of official communications concerning COVID-19. The highest correlations of the term ‘RKI’ with reported COVID-19 cases were found between lags of − 2 and − 12 days, meaning web searches were already performed from 2 to 12 days before case numbers increased. A similar pattern was seen for the term ‘corona’. Cross-correlations indicated that most searches on ‘protective mask’ were performed from 6 to 12 days after the peak of cases. The results for the term ‘protective mask’ indicate a degree of confusion in the population. This is supported by conflicting recommendations to wear face masks during the first wave. The relative search volumes could be a useful tool to provide timely and location-specific information on public attention for risk communication.

## Introduction

COVID-19 (Corona Virus Disease 2019) is caused by the Severe Acute Respiratory Syndrome Coronavirus (SARS-CoV-2). The global spread of the virus led to COVID-19 being classified as a pandemic in March 2020, affecting the lives of billions of people. At the time of writing, over 29 million confirmed cases and nearly 950,000 deaths have been reported worldwide^1^. In Germany, ca. 260,000 confirmed cases and 9400 deaths have been reported during the first wave^2^.

A distinctive feature of infectious diseases is that individual behaviour can also impact the health of others. Consequently, human behaviour plays a major role in the research and control of infectious diseases^3–5^. In the case of COVID-19, preventive measures to contain the spread of the virus and reduce the burden on health systems are highly relevant given its high secondary attack rate and outstanding deadly toll. In addition to government-imposed contact restrictions and other social distancing policies, a number of individual hygiene measures, i.e. regular hand washing and wearing masks, are recommended in order to contain the spread and to protect oneself^6^.

The risks and uncertainties of emerging infectious diseases can trigger a diverse array of emotional, cognitive, and behavioural responses and affect public behaviour in both constructive (e.g. adopting individual hygiene measures) and destructive manners (e.g. excessive use of health care services)^7–9^. Risk communication, defined as “the exchange of information among interested parties about the nature, magnitude, significance, or control of a risk”^10^, aims at increasing the capacity of the public to act as an effective response partner by encouraging constructive responses amongst the population, i.e. by adopting desired prevention measures^11,12^. Behaviour change can be induced through risk communication by presenting a threat and describing a behaviour change that can mitigate the threat^13^. For supporting effective risk communication it is important to understand how critical health risk information is disseminated and how the public accesses, processes and responds to this information^14^. Research on risk communication has already provided numerous insights thereto. For example, messages are more effective when delivered clear and simple, appeal to reason and emotion, and are tailored to the needs, values, cultural background and experiences of the target audience^13^. In addition, the characteristics of the entity sending a message are likewise important, for example, credibility of authority, transparency and perceived expertise influence the persuasiveness of the communication^15^.

Important prerequisites for effective communication, i.e. communication that leads to the desired constructive response, is described in the Protective Action Decision Model. According to this Model, the target population must (a) be exposed to the information, (b) pay attention to it and (c) understand it^16^. Measuring the degree of exposure, attention and comprehension of communications would usually require time-consuming and resource-intensive data collection methods. Particularly in the case of public attention, conventional data collection methods such as surveys are criticised for not being able to adequately capture the dynamic nature of public attention^17^. The vast amount of real-time information contained on the internet could open up innovative ways to assess public attention^17^. Web searches are not only a valuable resource for individuals seeking information, but also for the scientific community, since search queries contain geospatial and temporal data.

The Internet represents a common source for finding health-related information^18^. Numerous studies have shown how analysing web search queries can assist in describing and predicting outbreaks especially in the context of sparse data^19–21^. This approach has also been used for COVID-19 already^22,23^. However, web searches have also been analysed to describe public attention in relation to a public health issue. In the area of non-communicable diseases, studies can be found that examine public attention to preventive measures such as cancer screening or smoking cessation^24–27^. In the area of infectious diseases, studies mainly examine public attention to the disease itself^9,28–31^, but further examine attention to specific prevention measures^32–35^. Public interest in key institutions during a pandemic providing information on who is involved in risk communication, namely public health authorities, has not yet been studied.

The aim of this study is to combine these approaches and to investigate the usefulness of clustered web searches as an indicator of public attention to the disease itself and to prevention-relevant aspects in order to inform risk communication strategies in the context of the ongoing pandemic in Germany.

## Materials and methods

### Data sources

#### Officially reported cases

Daily incidence data of officially reported COVID-19 cases in Germany were obtained from the Robert Koch Institute (RKI) which is the national public health authority in Germany. Data for the first wave was used, which corresponds to the period from 15 February 2020 to 27 April 2020. Data are publicly available (https://npgeo-corona-npgeo-de.hub.arcgis.com/datasets/ef4b445a53c1406892257fe63129a8ea_0?geometry=-19.624%2C46.270%2C35.879%2C55.886). The daily number of new cases were retrieved for each of the 16 federal states in Germany separately as well as clustered national data.

#### Web search queries

Google Trends provides access to Internet search patterns by analysing web queries on the Google search browser and other affiliated Google sites. It provides a Relative Search Volume (RSV), i.e. the query share of a particular term for a given location and time, normalised by the highest query share of that term over the given time period. Data is presented on a relative scale from 0 to 100, with 0 representing no related queries and 100 representing the maximum number of related queries.

During the period of 15 February to 27 April, various German query terms used for seeking information on COVID-19 and its prevention measures were investigated. Search terms were collected and divided into the following thematically related groups: the virus or the disease itself, sources of information and preventive measures, which included hand hygiene and facial masks. After this, search terms of each group were jointly queried on Google Trends to identify the most frequently searched terms within a thematic group. Search terms related to the disease or virus, respectively were ‘corona’, ‘coronavirus’, ‘covid-19’ and ‘covid 19’. Search queries related to information sources were ‘corona podcast’, ‘corona info’, ‘corona hotline’, ‘Robert Koch Institut’, ‘RKI’, and ‘Gesundheitsamt’, whilst search queries for hand hygiene and masks were ‘Händewaschen’, ‘Hände waschen’, ‘Händedesinfektion’, ‘Händedesinfizieren’ and ‘Hände desinfizieren’, and ‘Mundschutz’, ‘Maske’, ‘Gesichtsmaske’, ‘Schutzmaske’, ‘Stoffmaske’, respectively*.* The most frequently searched terms were then selected. Finally, daily data from 15 February to 27 April 2020 on web searches for ‘RKI’, ‘Mundschutz’ (protective mask) and ‘corona’ were obtained separately from Google Trends (https://trends.google.com/trends/) on 01 May 2020. Data were retrieved for each federal state in Germany and nationally clustered for the whole country. Thereafter, data was downloaded in csv-format. Search queries related to hand hygiene were not suitable for further statistical analyses because the RSV only displayed extreme values around 0 or 100 with little variation in between. Therefore, this group of search queries was excluded from subsequent analyses.

#### Risk communication milestones

Important announcements in the period from 15 February to 27 April were retrieved from timelines published by the German Ministry of Health (MoH) (https://www.bundesgesundheitsministerium.de/coronavirus/chronik-coronavirus.html) and the World Health Organization (WHO) (https://www.who.int/news-room/detail/29-06-2020-covidtimeline). In addition, short reports during the same period from a major national public media network (https://www.tagesschau.de/faktenfinder/hintergrund/corona-chronik-pandemie-101.html) were screened.

### Data analysis

Statistical analyses were conducted using SPSS version 25 and R version 3.6.2. Line plots in combination with histograms, and cross-correlations were generated to assess the association between Google search trends and the officially reported COVID-19 cases.

To characterise time dependence between time-series from two different data sources, cross-correlations were used. The time dependence between two time-series is termed as lag and indicates the direction of the two time-series correlated. A lag of − 1 suggests that a peak in Google Trends precedes a peak in the officially reported COVID-19 cases by 1 day, and vice versa for positive lags.

Both time series were normalised to account for the different units and scales (RSV and absolute number of daily new cases). Thresholds for interpreting a correlation coefficient suggested by Hinkle et al. were applied^36^.

The risk communication milestones were reviewed based on their content and relevance for the present study by two independent reviewers. Disagreement was solved by discussion.

### Ethics declarations

Ethical approval and consent to participate were not necessary as the study was based on openly available aggregated data.

## Results

### Time trends

**Figure 1:**
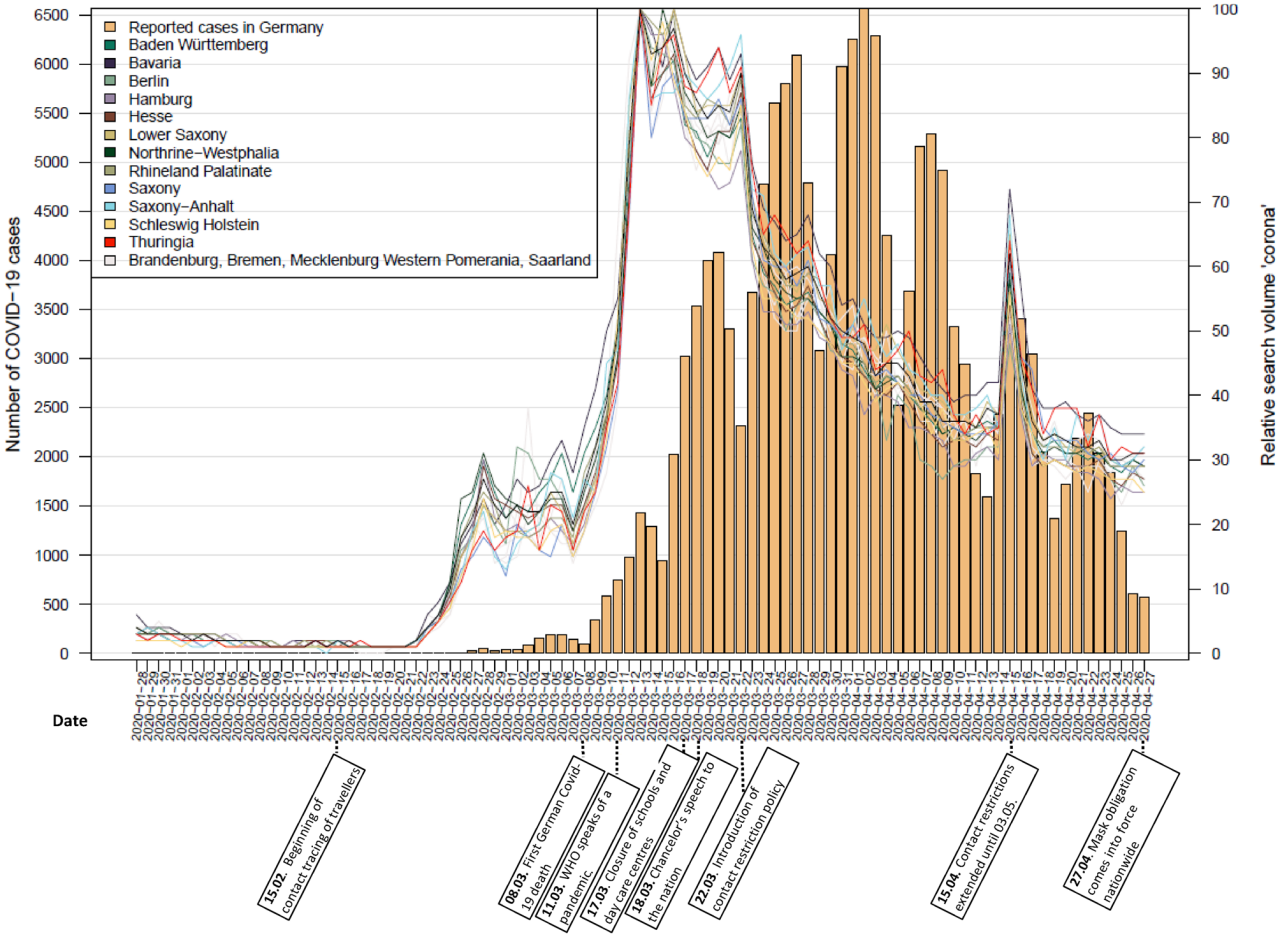
Nationally reported COVID-19 cases and RSV for the search term ‘corona’ at state level.

**Figure 2:**
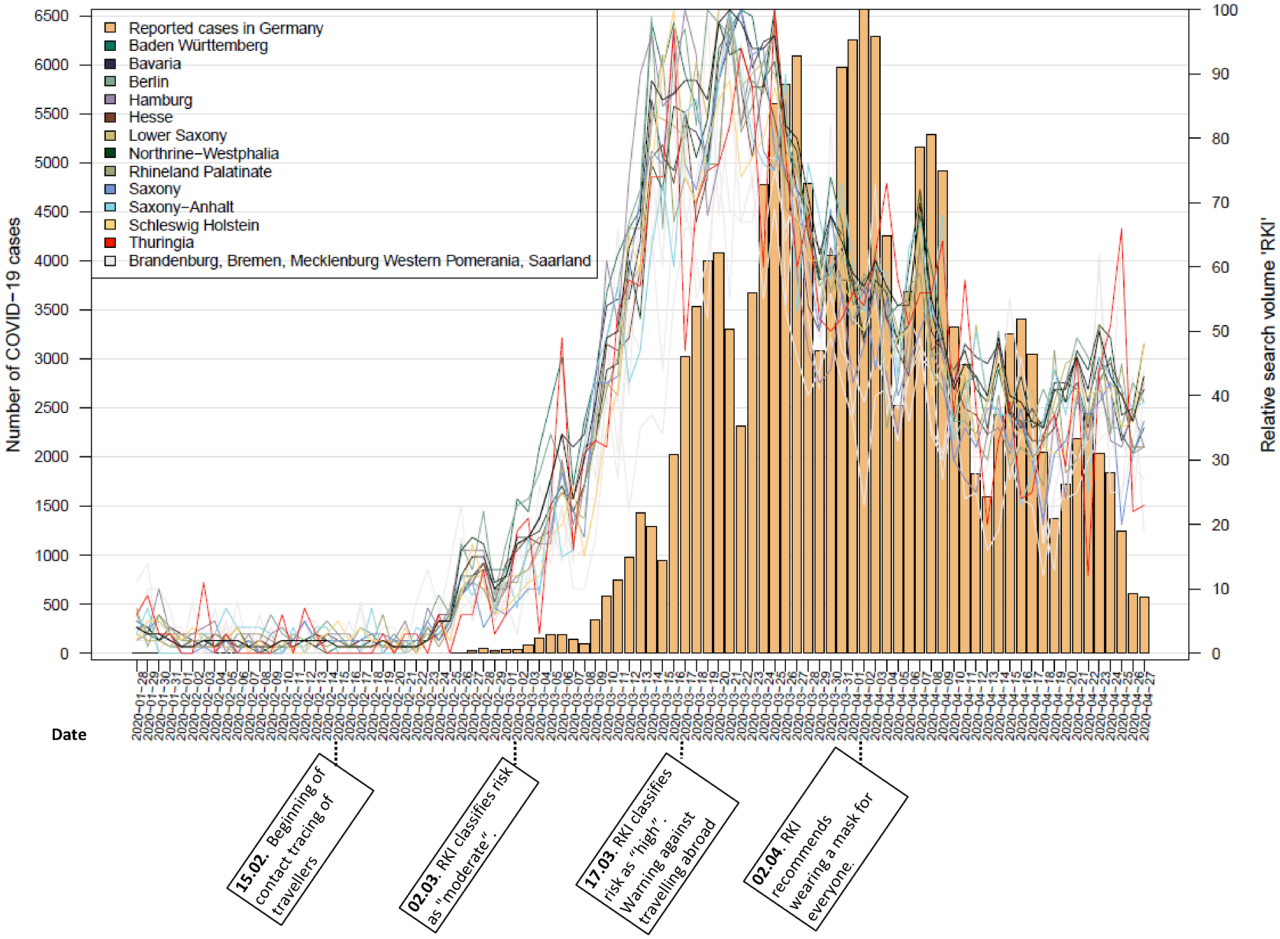
Nationally reported COVID-19 cases and RSV for the search term ‘RKI’ at state level.

**Figure 3:**
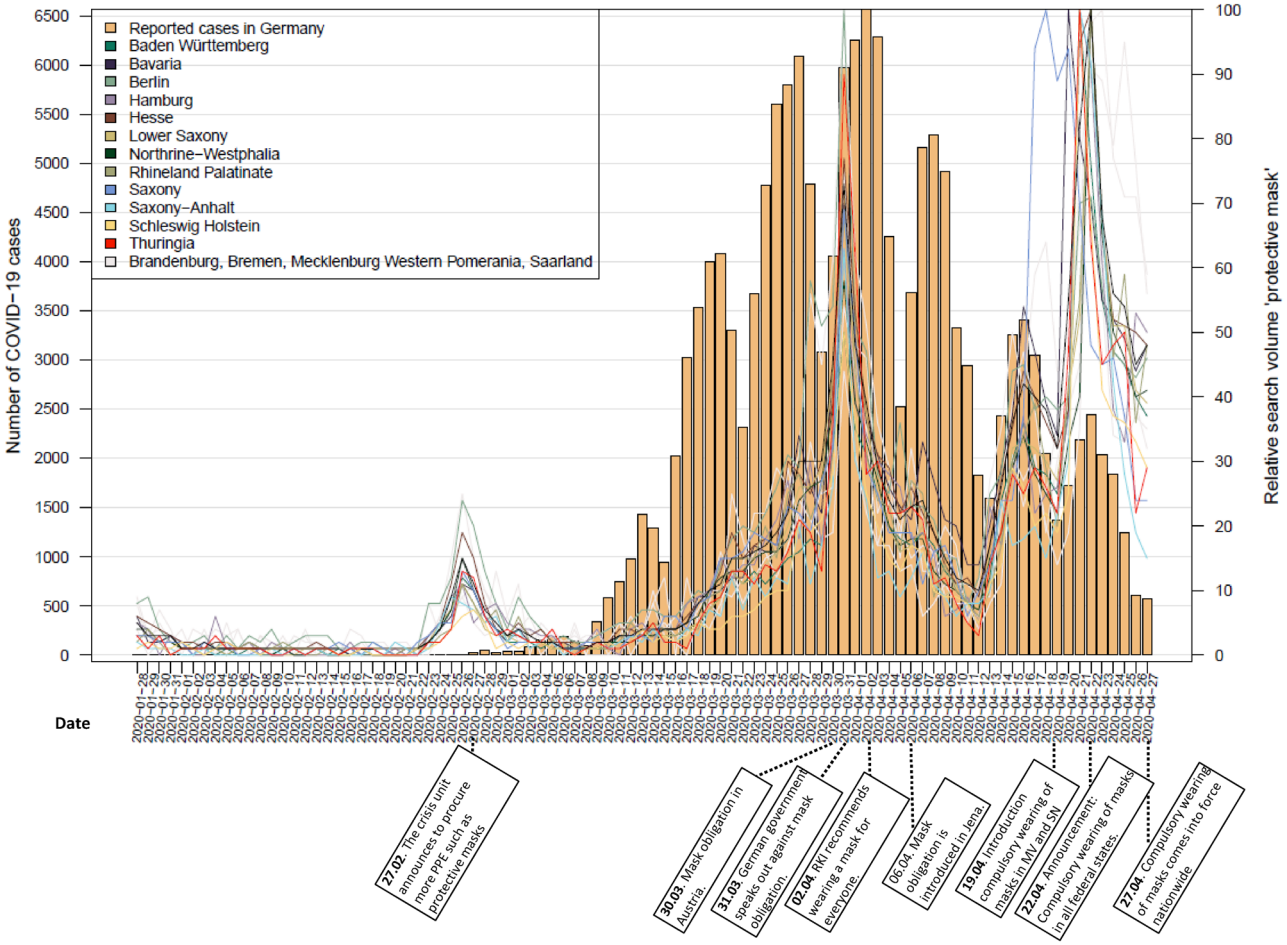
Nationally reported COVID-19 cases and RSV for the search term ‘protective mask’ at state level.

Histograms of officially reported cases on a national level in combination with line graphs of Google search queries for ‘RKI’, ‘corona’ and ‘protective masks’ at state level are shown in Figs. 1, 2 and 3. Most relevant communications and announcements are also presented in these figures. Figures 1 and 2 show that in all German federal states the RSV of the search terms ‘RKI’ and ‘corona’ reach their peak (mid-March) before the number of cases peak (beginning of April). The RSV of ‘corona’ shows a second peak of searches in mid-April. Meanwhile the RSV of ‘protective mask’ presents with three peaks with the highest peak at a point where the curve of confirmed cases is already flattening (end of April) (Fig. 3).

### Time-series cross-correlations

**Figure 4:**
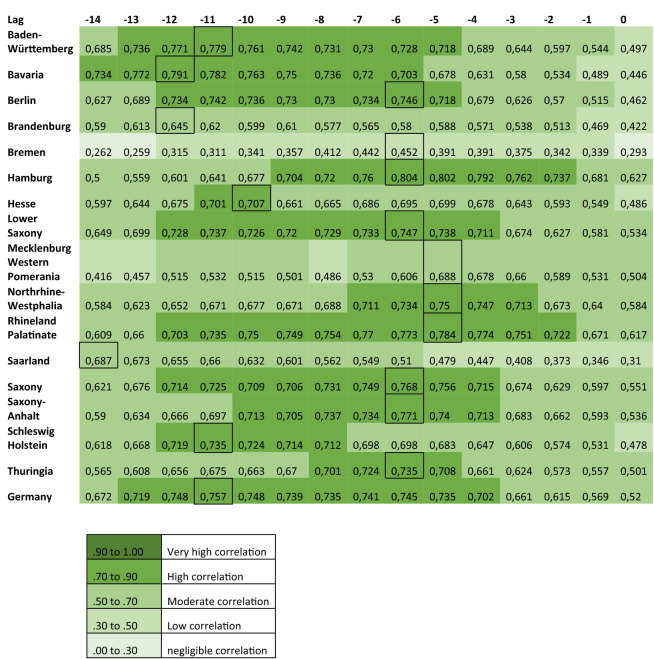
Cross-correlation coefficients displaying the linear association between the RSV of the term ‘corona’ and officially reported COVID-19 cases.

**Figure 5:**
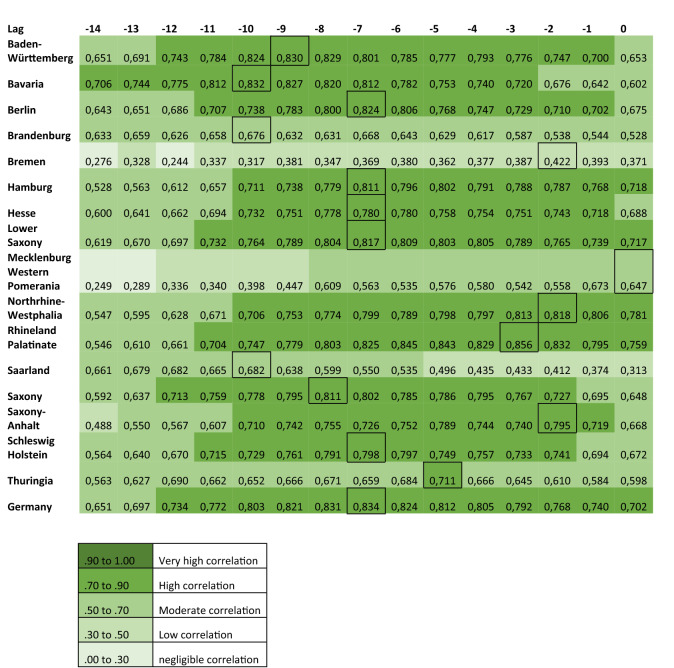
Cross-correlation coefficients displaying the linear association between the RSV of the term ‘RKI’ and officially reported COVID-19 cases.

**Figure 6:**
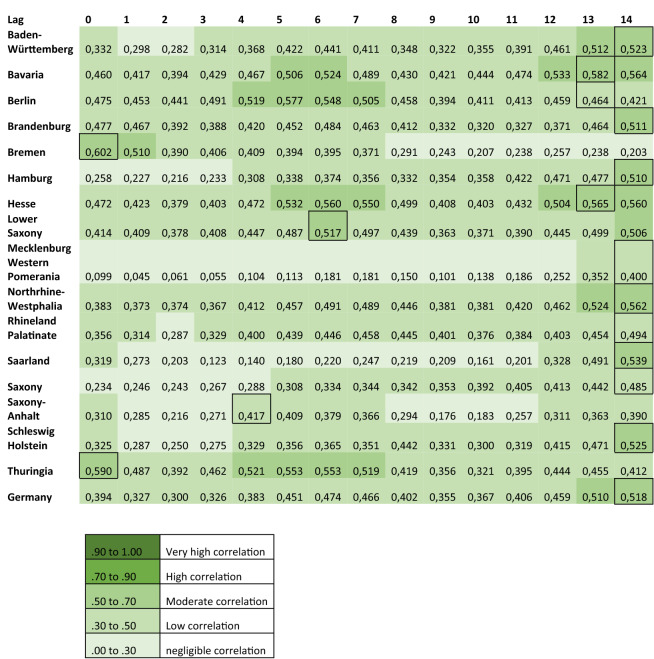
Cross-correlation coefficients displaying the linear association between the RSV of the term ‘protective mask’ and officially reported COVID-19 cases.

The linear association between officially reported COVID-19 cases and Google Trends patterns for the search terms ‘RKI’, ‘corona’ and ‘protective’ mask were assessed using cross-correlations, as shown in Figs. 4, 5 and 6.

Visual assessment of Figs. 4 and 5, which refer to the search terms ‘corona’ and ‘RKI’ respectively, reveals that the correlations are high in most federal states with the exception of Brandenburg, Bremen, Mecklenburg-Western Pomerania and Saarland. These federal states are described separately from the rest in the following.

For the group of federal states with high correlation, the RSV of the term ‘corona’ correlates highly with officially reported cases (r = 0.707 to r = 0.804) at lags between − 5 and − 12 days. In the same group of states, the RSV for ‘RKI’ and reported cases correlate highly (r = 0.711 to r = 0.856) when searches precede the reported cases between 2 and 10 days (range of lags: − 2 to − 10 days).

In the federal states Brandenburg, Bremen, Mecklenburg-Western Pomerania and Saarland only moderate correlations (r = 0.453 to r = 0.688) are seen between the RSV of the term ‘corona’ and reported cases. Correlations are highest when the searches precede the reported cases (range of lags: − 5 to 14 days). For these states, a similar trend can be observed for the term ‘RKI’ (r = 0.449 and r = 0.682; range of lags: − 10 to 0 days)*.*

A different picture results from the search term ‘protective mask’ (Fig. 6). In contrast to the other two search terms, the highest correlation coefficients (r = 0.400 to r = 0.602) result when reported cases precede the RSV for ‘protective mask’ by 5 to 14 days (range of lag + 5 to + 14 days).

### Risk communication milestones and time trends of search queries

**Figure 7:**
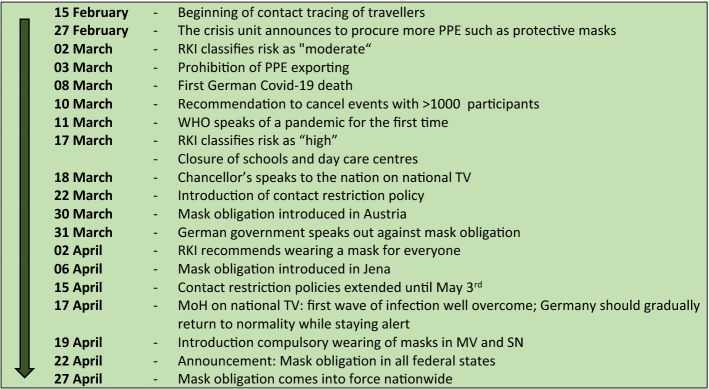
Timeline of communication milestones from 15 February to 27 April 2020.

Following the timeline of the data period (15 February to 27 April) chronologically (Fig. 7), an important announcement was made on 02 March when the RKI classified the risk as “moderate”. Just over a week later, on 11 March, COVID-19 was classified as a pandemic by WHO. During this period, searches for ‘RKI’ and ‘corona’ began to soar (Figs. 1, 2). On 13 March, most of the German states announced that they would temporarily close the schools. On 17 March, the RKI classified the risk as “high” and all schools and day care centres were closed. At that time, the search queries for the terms ‘corona’ and ‘RKI’ both reached their highest level (Figs. 1, 2). Five days later, on 22 March, the Federal Government and the federal states agreed on guidelines for restricting social contacts. Central elements were the prohibition of staying in public places with more than one other person not living in the same household, and the closure of restaurants, bars, and service providers with physical contact, such as hairdressers or massage studios. At this point, there was a renewed increase in search queries of the term ‘corona’. The RSV for ‘corona’ previously showed a decreasing trend.

On 31 March, the German government expressed its opposition to the obligation to wear masks as medical masks should be reserved for health care personnel. At this point, the search queries for the term ‘protective mask’ increased steeply in all federal states (Fig. 3). On 02 April, the RKI extended its recommendation regarding wearing masks. While wearing masks was previously recommended for people with respiratory diseases, they now recommended wearing masks in public places for the general population. A few days later (06 April), compulsory wearing of masks was introduced in Jena, a city in the federal state of Thuringia. In the following weeks, several federal states introduced compulsory wearing of masks, while there was no nationwide regulation. Meanwhile, it was announced on 15 April that the contact restrictions, announced on 22 March, would be extended until 03 May and that the schools would reopen on 04 May. The previously decreasing trend seen in the RSV of the term ‘corona’ showed a sudden increase at this point (Fig. 1). On 17 April, the Federal Minister of Health stated on national television that the first wave of infection had been well overcome and that the country should gradually return to normality while staying well-prepared and alert. On 22 April, a nationwide mask obligation was announced which came into force on 27 April. At this point, queries for ‘protective mask’ then reached their peak in the period under review (Fig. 3).

## Discussion

The findings show that different search terms related to the pandemic had different trends over the course of the first wave. While searches for the terms ‘RKI’ and ‘corona’ reached their peak before the number of cases peaked, the RSV of the term ‘protective mask’ peaked when the curve was already flattening.

The RSV for the term ‘RKI’ followed a similar trend as the RSV for the term ‘corona’, with relatively few searches until the end of February and most searches performed in mid-March with a subsequent decline in searches. At times of increasing attention to ‘corona’, attention to the national health authority ‘RKI’ also increased. This could indicate that the Institute is a trusted source of information for the public. This hypothesis is supported by data from the GESIS Panel Special Survey on the Coronavirus SARS-CoV-2 Outbreak in Germany. The data displays, that the RKI enjoys the greatest public trust in dealing with the pandemic compared to other institutions^37^.

The temporal relationship between the RSV of ‘corona’ and the reported cases suggests that the term has a certain predictive value that could be used in the context of sparse data. A study by Effenberger et al. examined this temporal relationship in different countries and also found that the peaks of web searches for the term ‘coronavirus’ preceded the peaks of the reported cases by 11.5 days on average^38^. However, visual examination of the data from this study also suggests that RSV is closely linked with risk communication milestones, such as categorisation of risk levels, public debates on appropriateness of measures, and policy announcements. The intention to include these milestones alongside the RSV of search queries and official case data is based on the potential influence of risk communication milestones on the way people respond and search for online information. Therefore, risk communication milestones may be considered important and meaningful in the visual representation along with the other two data sources.

The observation related to an increase in web searches on ‘RKI’ and ‘corona’ preceding the increase of reported cases was also described in a similar study that monitored search terms related to COVID-19 such as ‘corona’, ‘handwashing’ and ‘masks’. In contrast to this study, however, web searches for the terms ‘face mask’ and ‘surgical mask’ behaved in the same manner and were performed before most cases were reported^33^.

This leads to the question of why the search term ‘protective mask’ displayed a different trend in Germany. The long absence of a nationwide uniform obligation to wear a mask, while such obligations have already been introduced at a local level, could have led to confusion and an increased the need for information retrieval on this topic on the Internet. In addition, there have been conflicting recommendations from various institutions as to whether masks should be worn by the general public or primarily by healthcare professionals. This public debate and uncertainty are reflected in the RSV of the term ‘protective masks’.

The previous example attests how the RSV of search terms can help to assess the response to announcements and messages disseminated to the target audience. We were able to show that at a time when public attention for ‘corona’ was at its highest, relatively few people informed themselves about prevention measures such as wearing protective masks on the internet. Only when a nationwide obligation to wear masks in public areas was announced, did search queries for the term ‘protective mask’ reach their peak.

In retrospective, this is important information for the risk communication strategy. Clarity in communication, especially on the topic of masks, could have stimulated interest in masks at an earlier stage. It would have been desirable for the public to be made aware of this prevention measure at the peak of the first wave, or preferably earlier, so that it could then obtain information about this preventive measure on the Internet.

The results of this study are subject to some limitations that should be considered. First, the intentions behind the analysed web searches are unclear. Different motives such as seeking health information, updating oneself on current policies, or purchasing face masks might have led individuals to search for the terms and do not necessarily represent certain attitudes towards any preventive measures or the disease itself. Furthermore, it is known based on literature that disease-related web searches are subject to media-driven interest known as “celebrity phenomena”^39^, which refers to increased internet queries for certain terms that are temporarily popular in the media.

Another important limitation arises from the limited applicability of the analysis method in federal states with low absolute populations, as it was the case in Brandenburg, Bremen, Mecklenburg-Western Pomerania and Saarland. The results showed that correlations between the RSVs and reported cases were only from low to moderate in these states. It is therefore questionable to what extent the presented method can be applied in areas with a low population.

Some search terms that are highly relevant for disease prevention and personal protection, namely search queries related to hand hygiene, could not be included in the analysis due to their data distribution. It is assumed that a reduced search frequency resulted in a distribution of data showing only very low or very high values. Different reasons might explain why these terms were not searched for with more frequency, e.g. no need in the population to inform themselves about these topics. As a final limitation, it should be mentioned that RSVs do not provide information about the absolute number of web searches. Therefore, it is not possible to draw conclusions about the actual number of searches performed, but only about their trends over time.

Nevertheless, in this study we could present a novel tool for the fast assessment of public attention in times of an ongoing pandemic and attest how web searches can also inform risk communication strategies about the attention paid to a risk and different aspects of it.

## Conclusion

In the context of an ongoing pandemic using RSVs of key terms related to the disease can help to inform about the public attention. Results suggest that the data can also be used to assess the response to messages disseminated. The example of the protective masks showed how initial public controversies and contradictions were reflected in the search behaviour of the population, which only informed themselves about protective masks when the curve already flattened.

## Data Availability

The Google Trends dataset generated for the current study is available and can be replicated at: https://trends.google.com/trends/. Daily incidence data on officially reported COVID-19 cases are publicly available: https://npgeo-corona-npgeo-de.hub.arcgis.com/datasets/ef4b445a53c1406892257fe63129a8ea_0?geometry=-19.624%2C46.270%2C35.879%2C55.886.
